# Denoising of pediatric low dose abdominal CT using deep learning based algorithm

**DOI:** 10.1371/journal.pone.0260369

**Published:** 2022-01-21

**Authors:** Hyoung Suk Park, Kiwan Jeon, JeongEun Lee, Sun Kyoung You

**Affiliations:** 1 National Institute for Mathematical Sciences, Daejeon, Republic of Korea; 2 Department of Radiology, Chungnam National University College of Medicine, Daejeon, Republic of Korea; 3 Department of Radiology, Chungnam National University Hospital, Daejeon, Republic of Korea; Newcastle University, UNITED KINGDOM

## Abstract

**Objectives:**

To evaluate standard dose-like computed tomography (CT) images generated by a deep learning method, trained using unpaired low-dose CT (LDCT) and standard-dose CT (SDCT) images.

**Materials and methods:**

LDCT (80 kVp, 100 mAs, n = 83) and SDCT (120 kVp, 200 mAs, n = 42) images were divided into training (42 LDCT and 42 SDCT) and validation (41 LDCT) sets. A generative adversarial network framework was used to train unpaired datasets. The trained deep learning method generated virtual SDCT images (VIs) from the original LDCT images (OIs). To test the proposed method, LDCT images (80 kVp, 262 mAs, n = 33) were collected from another CT scanner using iterative reconstruction (IR). Image analyses were performed to evaluate the qualities of VIs in the validation set and to compare the performance of deep learning and IR in the test set.

**Results:**

The noise of the VIs was the lowest in both validation and test sets (all p<0.001). The mean CT number of the VIs for the portal vein and liver was lower than that of OIs in both validation and test sets (all p<0.001) and was similar to those of SDCT. The contrast-to-noise ratio of portal vein and the signal-to-noise ratio (SNR) of portal vein and liver of VIs were higher than those of SDCT (all p<0.05). The SNR of VIs in test sets was the highest among three images.

**Conclusion:**

The deep learning method trained by unpaired datasets could reduce noise of LDCT images and showed similar performance to SAFIRE. It can be applied to LDCT images of older CT scanners without IR.

## Introduction

Demand for radiation dose reduction is growing as the use of computed tomography (CT) for pediatric patients increases [1, 2]. Radiation dose reduction is commonly achieved by reducing the X-ray tube current (milliampere-seconds; mAs) or tube voltage (kilovoltage peak; kVp) [2]. However, low-dose CT (LDCT) images reconstructed using the conventional filtered back projection method [3] suffer from excessive quantum noise, resulting in degradation of diagnostic performance. With recent advances in CT technology, various commercial iterative reconstruction (IR) methods have been proposed and have demonstrated the potential to improve the quality of the images reconstructed from low-dose scans [4–6]. An IR apparatus is usually mounted on relatively new CT scanners, and hence IR reconstructions are not available on older CT scanners.

Deep learning, a type of machine learning [7], has been recently proposed for CT dose reduction and has shown the potential to reduce noise artifacts [8–10]. Most of these approaches are based on learning the relationship between LDCT images and standard-dose CT (SDCT) images by using a pair of low-dose and high-dose CT images. However, obtaining two scans with low-dose and standard-dose protocols simultaneously is often not feasible in real medical imaging practices.

To overcome the difficulty of preparing a paired dataset, we consider adopting the generative adversarial network (GAN) [11] that can learn the translation mapping from a source domain to another target domain [12, 13]. The GAN is a framework consisting of two competing neural networks: a generator and a discriminator. The generator attempts to generate samples in the target domain, while the discriminator attempts to distinguish between the samples generated by the generator and real samples in the target domain. By competing with each other, the generator enables the generation of samples in the target domain. In medical imaging, different variants of GANs have been applied to LDCT image denoising [14–16], in which the generator maps LDCT images to SDCT images. However, paired datasets were used to train these networks. Recently, some studies have explored the feasibility of applying a GAN approach for paired but spatially misaligned datasets [17], or unpaired datasets [18–22].

The purpose of this study is to determine whether a deep learning algorithm trained using unpaired LDCT and SDCT images can generate virtual SDCT images in a clinical environment. In this study, we adopted the approach described in a previous study [19] to generate the virtual SDCT images (VIs) from the original LDCT images (OIs) and called it virtual image generative adversarial network (VIGAN). This study was performed in two stages. First, we trained the VIGAN using unpaired datasets, which consisted of LDCT (80 kVp) and SDCT (120 kVp) images collected from various pediatric abdominal CT images. Second, we evaluated the ability of the trained network to generate VIs. Compared with the previous study [19], the main contributions of this study are as follows: 1) the performance of VIGAN was compared with that of commercial software (SAFIRE); 2) its feasibility was investigated on a relatively large clinical dataset, and was evaluated through an external clinical dataset (i.e., datasets acquired using a CT scanner not used for training); 3) further analyses (e.g., noise power spectrum and qualitative analyses) were performed for image quality evaluation.

## Materials and methods

This study was approved by the institutional review board of our institution, and the requirement for informed consent was waived.

### Data preparation

#### Dataset preparation

All the CT images used for training and validation of VIGAN were obtained using a 64-channel multidetector CT scanner (Sensation 64; Siemens Healthcare, Forchheim, Germany) with a tube current modulation program (CARE Dose4D) and reconstructed using FBP. This CT scanner does not have an automatic tube voltage selection program or an IR method. LDCT (fixed tube voltage of 80 kVp with 100 mAs reference tube current) from after September 2017 and SDCT (fixed tube voltage of 120 kVp with 200 mAs reference tube current) before September 2017 were used for training and validation of VIGAN, respectively.

**Fig 1:**
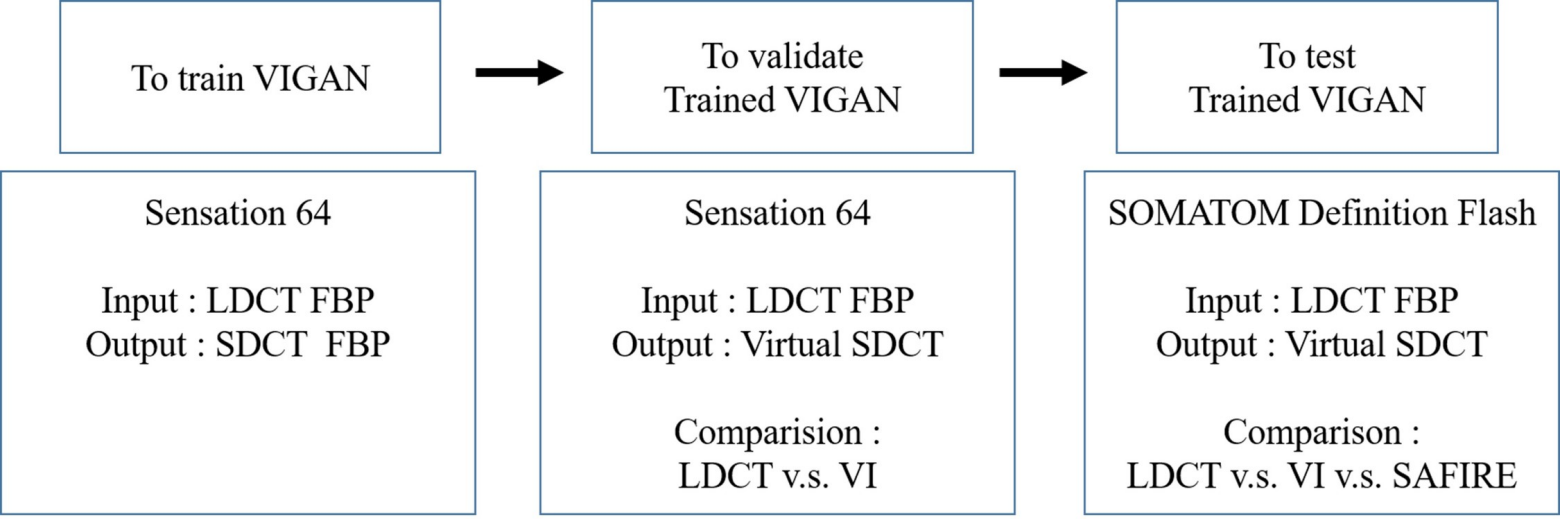
Flow diagram of study. * VIGAN = generative adversarial network for virtual standard dose CT image, LDCT = low dose computed tomography, SDCT = standard dose CT, VI = virtual image, SAFIRE = sinogram affirmed iterative reconstruction.

To test the trained VIGAN, we obtained LDCT (80 kVp with 262 mAs reference tube current) images taken in 2018 from another CT scanner (SOMATOM Definition Flash; Siemens Healthcare, Forchheim, Germany), which had an automatic tube voltage selection program and used an IR method. CT images were reconstructed by FBP and sinogram affirmed IR (SAFIRE, I30f, strength level 3). A summary of the processes used in our study is depicted in Fig 1. We prepared 20 DICOM format files covering the liver around the portal vein for each patient.

Finally, we collected LDCT and SDCT images of 42 patients for training, LDCT images of 41 patients for validation, and LDCT images of 33 patients for testing. A total of 840 LDCT and 840 SDCT images were used to train VIGAN, 820 LDCT images were used for validation, and 660 LDCT images were used for test.

#### Radiation dose measurement

The CT dose index volume (CTDIvol, mGy) and the dose-length product (DLP, mGy·cm) were recorded. The effective dose (ED, mSv) was calculated as ED = DLP × K (tissue-weighting factors for abdomen; variable according to tube voltage and age).

### GAN-based virtual standard-dose image generation model

**Fig 2:**
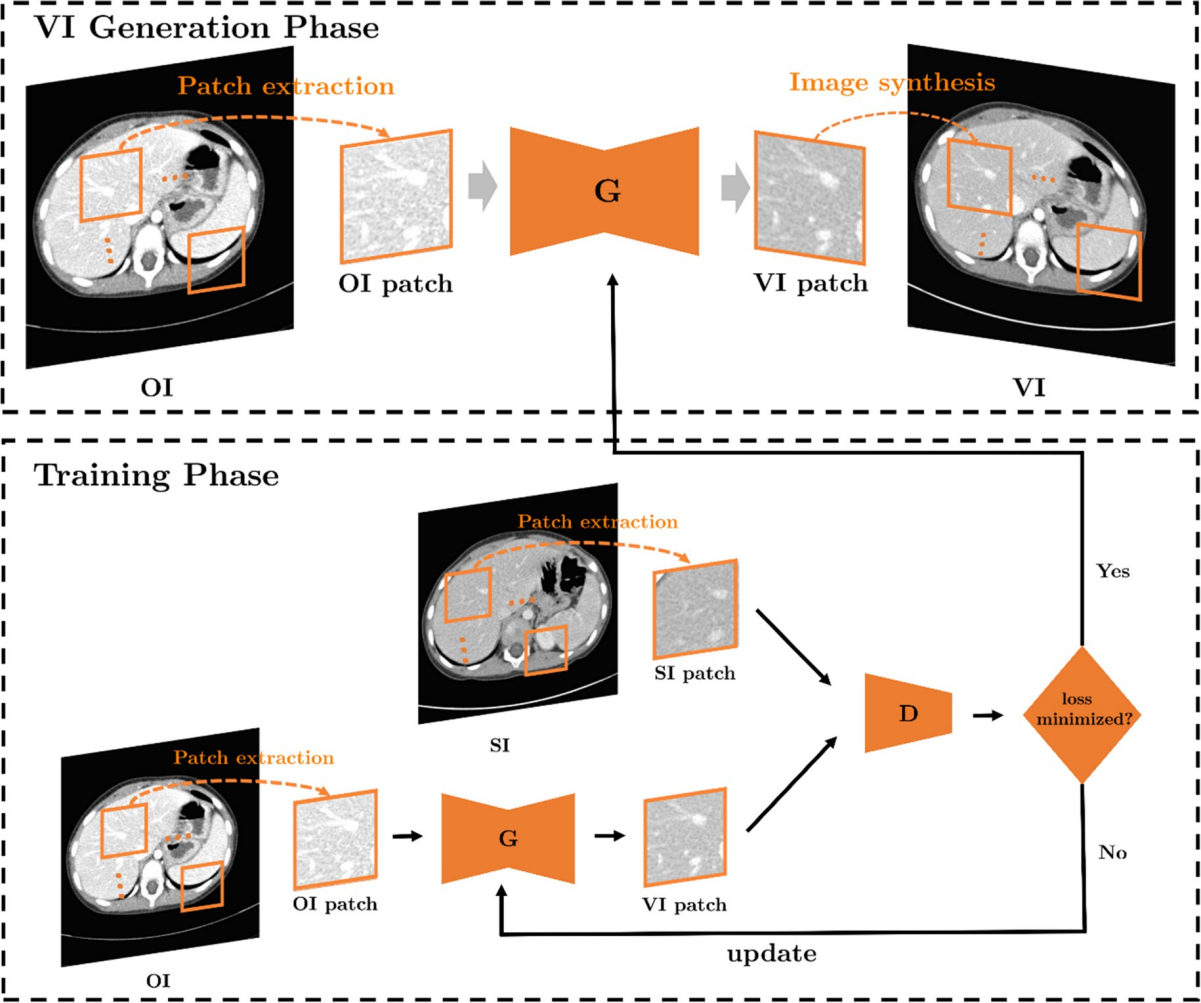
Schematic of the VIGAN. The VI generation is performed in a patch-by-patch manner by the trained generator. G = generator, D = discriminator. VI = virtual standard-dose CT images, OI = original low-dose CT image, SI = real standard-dose CT image.

The VIGAN consisted of three main steps. First, given an OI image of size 512*512, patches of size 128*128 were extracted with strides of 32 in each direction of the image domain. Second, the VI patches were generated from extracted OI patches using the proposed network, which will be explained below. Finally, the patches in the OI were replaced by the VI patches to obtain the VI. Here, the pixel values in the overlapping region of the patches were averaged. Fig 2 provides a schematic of the VIGAN.

The proposed network was mainly based on the GANs [11] that consist of two parts: generator and discriminator. The generator tries to generate VI patches that look similar to real SDCT patches, whereas the discriminator tries to distinguish between VI and real SDCT patches.

The pixel-wise loss between VI patches and OI patches was added to the network. This allows the VI patches to retain the morphological information of OI patches while reducing the noise. To improve the training stability and quality of patches for the GAN, a least-squares loss function was used as a discriminator classifier [23]. The mathematical model is described in detail in the S1 File.

**Fig 3:**
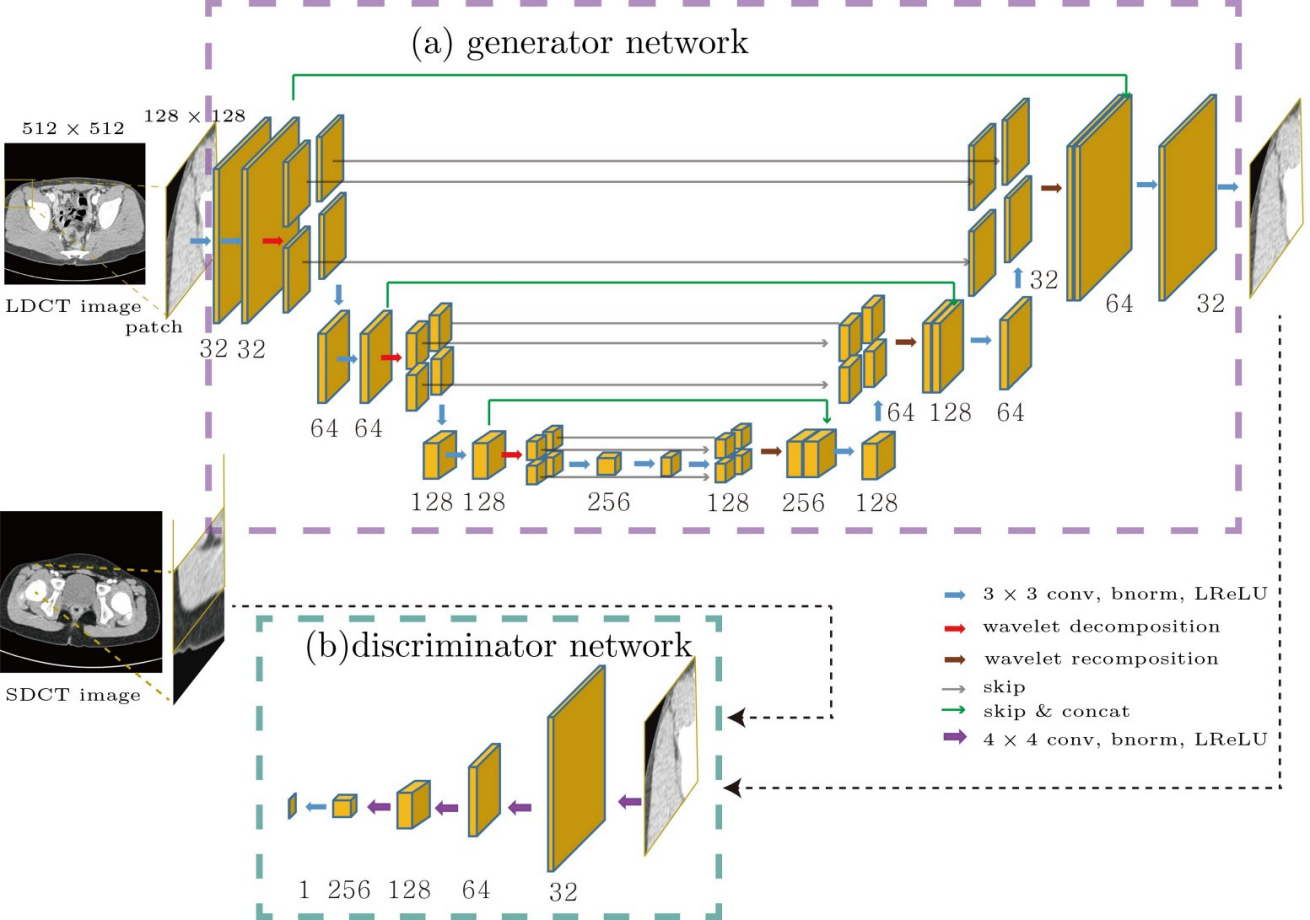
Network architectures of the VIGAN: generator (a) and discriminator (b). The number below the yellow box denotes the number of features used for training. VIGAN = generative adversarial network for virtual standard dose CT image generation. bnorm = batch normalization, conv = convolution, LReLU = leaky rectified linear unit, concat = concatenation.

The architecture of the generator and discriminator of the proposed network is illustrated in Fig 3. For the generator, we adopted a deep convolutional framelet [24] that consisted of a contracting path and an expansive path with skipped connection and concatenation layers. Each step of the contracting and expansive path contained two convolutions with a 3×3 window, each of which was followed by batch normalization [25] and a leaky rectified linear unit (ReLU) [26]. Next, the 2D Haar wavelet decomposition and recomposition [27] were used for downsampling and upsampling, respectively. The high-pass filters from the wavelet decomposition skipped to the expansive path, whereas the low-pass filters were concatenated with the features in the contracting path during the same step. Finally, an additional 1×1 convolution layer was added to generate a grayscale output image. Every convolution in our network was performed with zero padding to match the sizes of the input and output images. In the adversarial architecture, the discriminator contained four convolutions with a 4×4 window and strides of 2 in each direction of the domain, each followed by batch normalization and a leaky ReLU with a slope of 0.2. After the last layer, a 1×1 convolution layer was added to generate 1-D output data.

**Fig 4:**
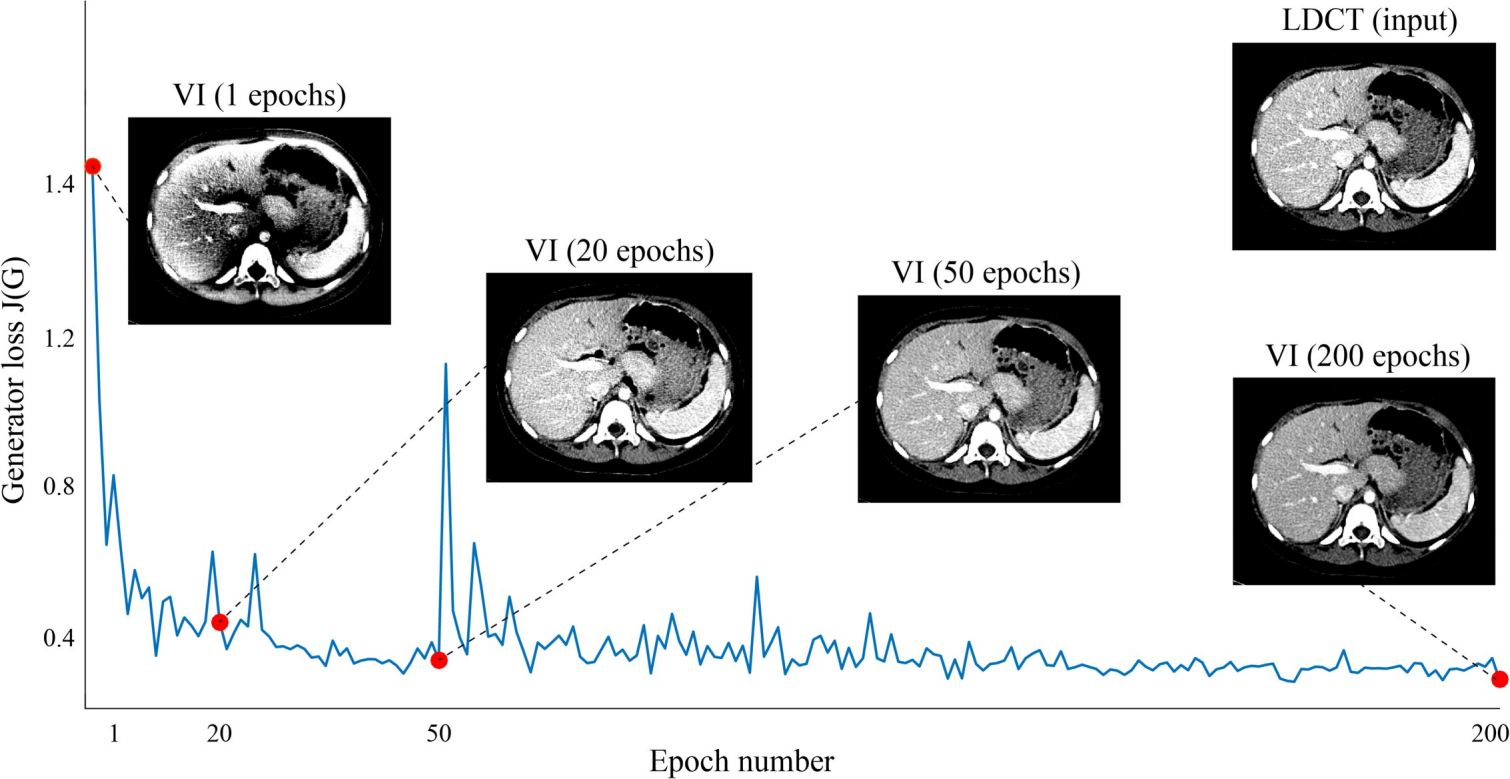
Graph of generator loss over the number of epochs, and the generated virtual SDCT images (VIs) at each epoch during the training process. (See Eq. (2) in the S1 File section for a mathematical expression of the generator loss J(G)).

As suggested by Park et al. [19], the proposed model was minimized using an Adam optimizer [28] with a learning rate of 0.0002 and mini-batch size of 40, and 200 epochs were utilized for training. The training was implemented using TensorFlow [29] on a GPU (NVIDIA, Titan Xp. 12GB) system. It required approximately one day to train our network. The network weights were initialized following a Gaussian distribution with a mean of 0 and a standard deviation of 0.01. Fig 4 shows the graph of generator loss over the number of epochs, as well as the generated VIs at each epoch during the training process.

### Data analysis

#### Quantitative analysis of virtual images

OIs and VIs were analyzed by a board-certified radiologist (S.K.Y., with 6 years of experience in pediatric radiology). Each DICOM file was displayed on a picture archiving and communication system workstation with soft tissue window settings (width, 250 HU; level, 125 HU). The mean CT attenuation was measured by manually placing a round region of interest (ROI) in each organ (portal vein, liver parenchyma, and paraspinal muscles). All measurements were performed at the main portal vein level. The size and shape of each ROI was kept constant in each patient. We used the copy and paste function to place ROIs in exactly the same location on the OI and VI. The attenuation of the portal vein was measured at the main portal vein using a single ROI; that of the liver was recorded using the mean of four ROIs by avoiding the inhomogeneous area and vessels; and that of the paraspinal muscle was recorded using the mean of the ROIs measured on both sides. The mean standard deviation for the paraspinal muscles was measured as image noise (SDn).

The contrast-to noise ratio (CNR) and signal-to-noise ratio (SNR) were calculated using the following equations: CNR = (ROIo–ROIm) / SDn and SNR = ROIo / SDn, where ROIo is the attenuation of the organ and ROIm is the attenuation of the paraspinal muscle.

The image quality was further evaluated using the noise power spectrum (NPS) [30] which represents the properties of image noise. For each image, multiple patches with a size of 32×32 were selected from homogeneous regions of the liver. The selected patches were normalized to their mean intensities and were used to calculate the NPS. The smaller the area under the curve (AUC) and peak frequency (i.e., the frequency at which the NPS has the maximum magnitude) of the NPS curve, the lower the noise level and image sharpness, respectively [31].

#### Qualitative analysis of virtual images

The qualitative analysis was performed independently by two board-certified radiologists (S.K.Y., with 6 years of experience in pediatric radiology, and J.E.L., with 8 years of experience in abdominal radiology). The readers assessed the image contrast, image noise, and overall image quality, by using a five-point scoring system. Before starting the subjective analysis, the two reviewers defined the assessment scale for the qualitative analysis of each item by consensus.

The five-point scale used to score the enhancement of the liver and portal vein is as follows: 1: very poor, 2: suboptimal, 3: acceptable, 4: above average, and 5: excellent. The five-point scale employed to assess the image noise is as follows: 1: unacceptable noise, 2: above-average noise, 3: average noise on an acceptable image, 4: less-than-average noise, and 5: minimum or no image noise. Finally, the five-point scale used to describe the overall image quality is as follows: 1: unacceptable diagnostic image quality, 2: sub-diagnostic, 3: average, 4: better than average, and 5: excellent.

#### Image distinction possibility

We also evaluated the “image distinction possibility” by assessing whether the reviewers could distinguish between SDCT and virtual SDCT by visual assessment. Two readers (H.S.K, and H.J.), who had not previously reviewed the virtual SDCT, reviewed the CT images in random order and determined whether the images were real or virtual.

#### Statistical analysis

The data were analyzed using IBM SPSS Statistics for Windows (Version 22.0., IBM Corp., Armonk, NY) and MedCalc (version 17.2, Mariakerke, Belgium). The statistical significance was defined as p < 0.05. The one-way analysis of variance (ANOVA) with a Tukey multiple-comparison post-hoc test was used to compare the linear measurements among the three groups. The significance levels of the post hoc tests were set at p < 0.016 to rectify the alpha error associated with multiple comparisons. A student’s t-test was used performed to assess the differences between the two groups and a p-value of <0.05 was considered statistically significant. The Cohen’s kappa statistic was used to assess the degree of inter-observer agreement of the qualitative analysis. The weighted kappa value was interpreted as follows: 0.81–1.00: excellent agreement, 0.61–0.80: substantial agreement, 0.41–0.60: moderate agreement, 0.21–0.40: fair agreement, and <0.20: poor agreement.

## Results

### Patients and radiation dose

The characteristics of the patients are summarized in Table 1. There were no significant differences in the sex and age of the patients included in the training and test datasets. The mean CTDIvol, DLP, and ED of LDCT were statistically lower than those of SDCT by 36.6%, 32.5%, and 31.8%, respectively.

**Table 1:** Patient characteristics and radiation dose.

	Training set		Validation set	p-value	Dose reduction*** (%)
	Standard-dose CT (120 kVp, n = 42)	Low-dose CT (80 kVp, n = 42)	Low-dose CT (80 kVp, n = 41)		
Sex (boys/girls)	24/18	31/11	24/17	0.21*	
Age (years)	6.2±2.2	7.2±2.5	7.4±2.2	0.59	
CTDI_vol_ (mGy)	4.1±1.1	2.6±0.5	2.5±0.4	< 0.001**	36.6
DLP (mGy·cm)	156.2±54.3	105.4±30.0	98.9 ± 22.9	< 0.001**	32.5
ED (mSv)	4.4±1.1	3.0±0.6	2.8±0.5	< 0.001**	31.8

* Pearson chi-square.

** Differences between standard dose CT and low dose CT from ANOVA test.

*** Calculated in training datasets between standard dose CT and low dose CT.

### Quantitative analysis of validation set

**Fig 5:**
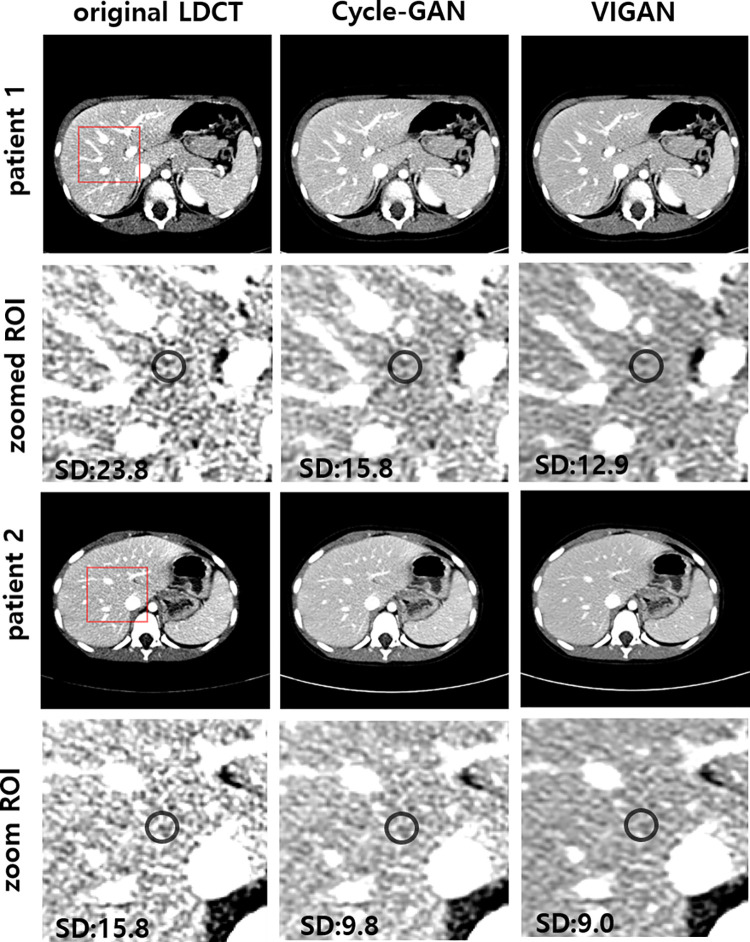
Images of original LDCT, Cycle-GAN, and VIGAN for validation set. Second and fourth rows show zoomed regions-of-interests marked with red rectangles in the images of the first and third rows. VIGAN achieves the lowest image noise (i.e., standard deviation(SD)) compared to Cycle-GAN and original LDCT. WL/WW = 115/250 for original LDCT. WL/WW = 85/250 for Cycle-GAN and VIGAN.

The CT numbers, image noise, CNRs, and SNRs of the OIs, Cycle-GAN, VIs, and SDCT are summarized in Table 2. The image noise of VIs was the lowest among those in validation set (p < 0.001, Fig 5). The mean CT numbers of the portal vein, liver, and paraspinal muscles of OIs were the highest among those in validation set (p < 0.001). The mean CNR of the portal vein and the mean CNR and SNR of the liver of VIs were higher than those of Cycle-GAN (all p<0.016). Based on a comparison of the results of of VIs and SDCT of training set, image noise of VIs was lower than those of SDCT (p = 0.006). There was no statistically significant difference of mean CT numbers of the portal vein, liver, and paraspinal muscles between VIs and SDCT(all p>0.05). The CNR of portal vein and the SNR of portal vein and liver of VIs were higher than those of SDCT (all p<0.05).

**Table 2:** Quantitative image analysis of training and validation dataset.

	OI of Validation dataset (n = 41)	Cycle-GAN of Validation dataset (n = 41)	VI of Validation dataset (n = 41)	SDCT of Training dataset (n = 42)	p-value of ANOVA -test among Validation dataset	OI v.s. Cycle-GAN	OI v.s. VI	Cycle-GAN v.s. VI	p-value of t-test between VI and SDCT
Image noise	14.2 ± 6.3	10.8 ± 2.1	9.4 ± 1.9	10.6 ± 2.5	< 0.001	< 0.001	< 0.001	0.008	0.006
CT number (mean±SD)									
Portal vein	247.3 ± 47.2	194.4 ± 30.8	199.5 ± 33.6	187.6 ± 23.4	< 0.001	< 0.001	< 0.001	0.82	0.72
Liver	145.6 ± 42.2	118.2 ± 15.9	119.2 ± 12.0	120.7 ± 12.6	< 0.001	< 0.001	< 0.001	0.967	0.561
Paraspinal muscle	75.8 ± 9.7	71.4 ± 6.2	69.8 ± 5.7	70.7 ± 5.7	0.001	0.021	0.001	0.582	0.425
CNR (mean±SD)									
Portal vein	13.1 ± 4.7	11.6 ± 3.4	14.2 ± 4.5	11.6 ± 4.7	0.024	0.139	0.640	0.016	0.001
Liver	5.3 ± 3.9	4.4 ± 1.7	5.4±1.7	5.0 ± 2.0	0.162	0.420	0.448	0.039	0.097
SNR (mean±SD)									
Portal vein	18.9 ± 5.4	18.5 ± 4.4	21.9 ± 5.5	18.6 ± 6.4	0.005	0.731	0.059	0.008	0.001
Liver	11.1 ± 4.4	11.3 ± 2.7	13.1 ± 2.9	12.0 ± 3.7	0.017	0.779	0.001	0.008	0.018

* OI; original image, VI; virtual image, SDCT; standard dose CT.

### Quantitative analysis of test set

**Fig 6:**
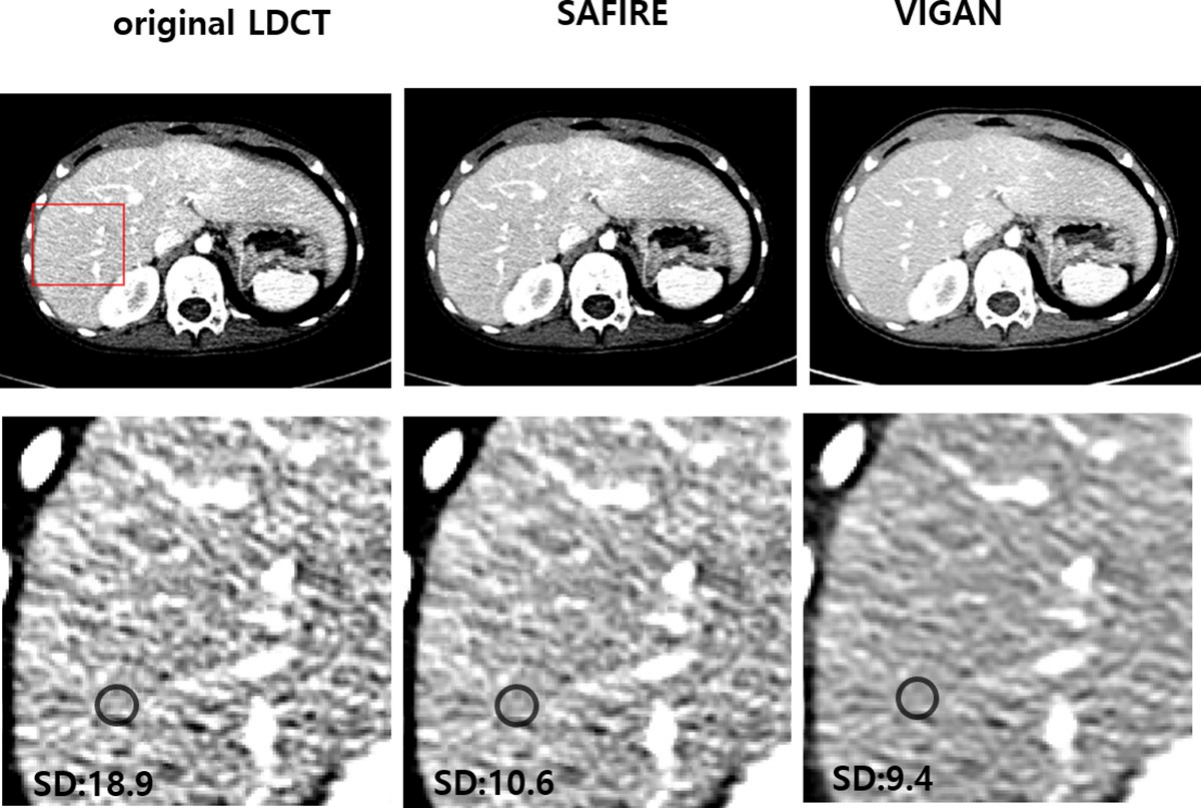
Images of original LDCT, SAFIRE, and VIGAN for test dataset. The second row shows zoomed regions-of-interests marked with the red rectangle in the images of the first row. The image noise (i.e., standard deviation(SD)) of VIGAN was lower than that of original LDCT and similar to that of SAFIRE. WL/WW = 125/230 for original LDCT and SAFIRE. WL/WW = 75/230 for VIGAN.

**Fig 7:**
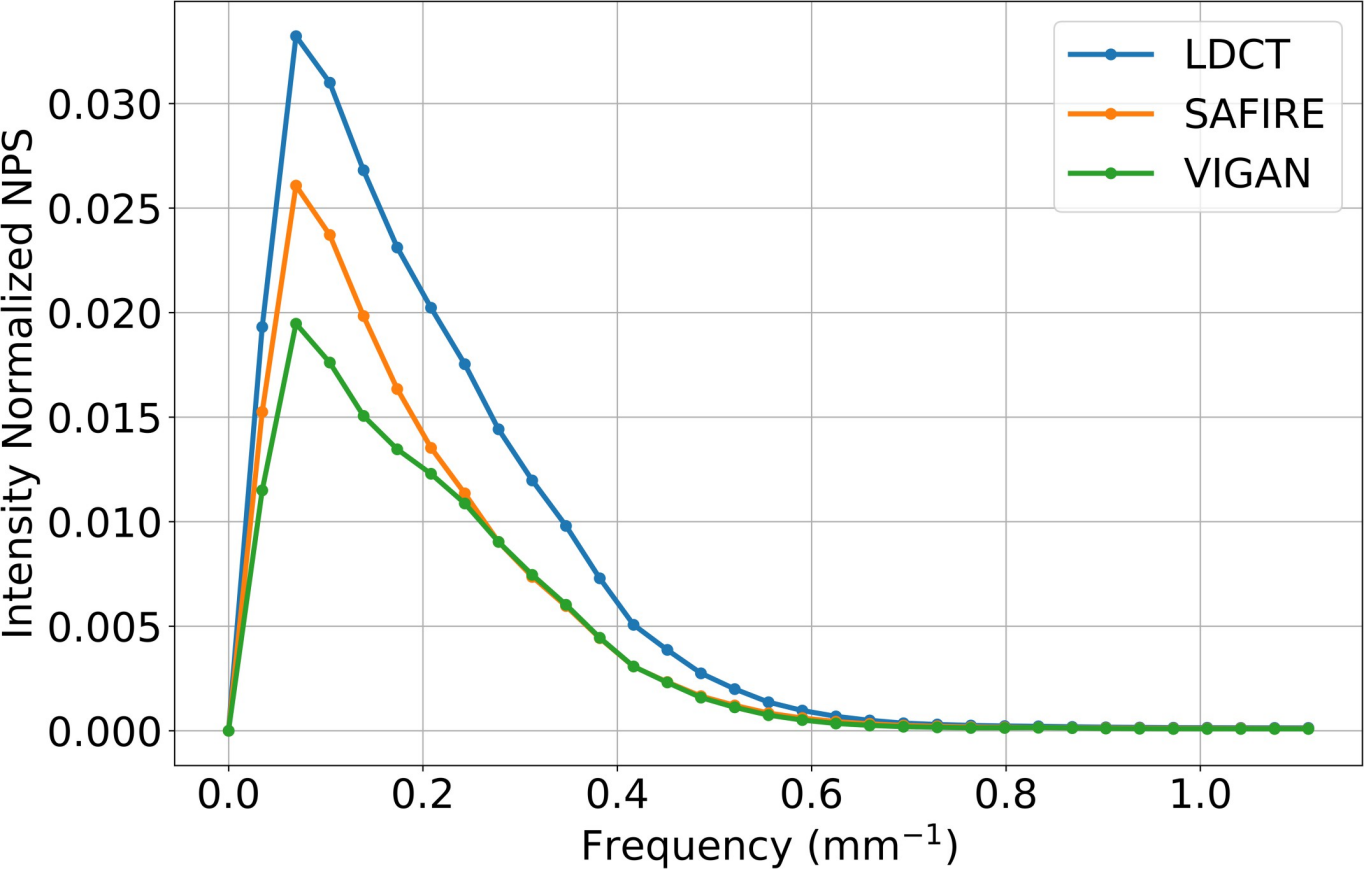
Comparison of intensity normalized NPS curves among original LDCT, SAFIRE, and VIGAN.

The CT numbers, image noise, CNRs, and SNRs of the OIs, SAFIRE, and VIs are summarized in Table 3. The mean CT numbers of VIS in the portal vein, liver, and paraspinal muscles were lower than those of OIs and SAFIRE (p < 0.001). The mean image noise of the VIs was the lowest among the three images (p < 0.001, Fig 6). In post-hoc test, there was statistically significant difference in image noise between the OI and SAFIRE, and between the OI and the VI (p = 0.011, p < 0.001, respectively). However, there was no statistically significant difference in image noise between the VI and SAFIRE (p = 0.059). The mean CNR of VIs of the portal vein and liver were higher than OIs and similar to SAFIRE, without any significant statistical difference. The mean SNR of VI of the portal vein and liver were highest among the three images (p < 0.006 and 0.003, respectively). In the post-hoc test, there was a significant difference only between the OI and the VI (p = 0.007, 0.003, respectively). Fig 7 shows the NPS curves for original LDCT, SAFIRE, and VIGAN. As shown in the figure, the AUC of VIGAN was lower than that of SAFIRE and original LDCT, however, the peak frequency was the same. This results indicate that VIGAN achieves better noise reduction while maintaining an adequate image sharpness compared with SAFIRE and original LDCT.

**Table 3:** Quantitative image analysis test data.

	OI	SAFIRE	VI	p-value			
	(n = 33)	(n = 33)	(n = 33)		OI v.s.SAFIRE	OI v.s. VI	SAFIRE v.s. VI
Image noise (mean±SD)	12.4 ± 5.0	9.5 ± 4	7.1 ± 2.7	< 0.001	0.011	< 0.001	0.059
CT number (mean±SD)							
Portal vein	248.9 ± 50.9	247.8 ±48.5	200.6 ± 45.5	< 0.001	0.995	< 0.001	< 0.001
Liver	139.8 ± 24.3	140. ± 24.4	118.3 ± 14.4	< 0.001	1	< 0.001	< 0.001
Paraspinal muscle	71.2 ± 10.3	71.5 ± 9.3	66 ± 7.8	0.027	0.99	0.061	0.044
CNR (mean±SD)							
Portal vein	16.2± 7.5	21.2± 9.8	21.2± 10.1	0.04	0.075	0.077	1
Liver	6.4± 3.7	8.5± 5.0	8.5± 4.3	0.1	0.154	0.153	1
SNR (mean±SD)							
Portal vein	22.9± 9.3	30.1± 12.2	31.9± 13.0	0.006	0.038	0.007	0.804
Liver	13.1± 5.7	17.3± 7.6	19.1± 7.9	0.003	0.054	0.003	0.554

* OI; original image, VI; virtual image.

### Qualitative analysis of validation set

The results of the qualitative analysis performed by the two readers are presented in Table 4. The inter-observer agreement was substantial to excellent because the weighted kappa values ranged from 0.68 to 0.93. The image contrast (enhancement of the liver and portal vein) and the overall image quality of the two groups were not significantly different (all *p* > 0.05). The image noise of VIs was estimated to be less than that of OIs (*p* < 0.001). Both OIs and VIs were assigned more than 3 points, indicating "average noise in an acceptable image."

**Table 4:** Qualitative image analysis for validation dataset.

	OI of validataion dataset	VI of validation dataset	p-value	k-value
**Enhancement of liver and portal vein**				
Reader 1	3.9±0.4	3.8±0.4	0.28	0.93
Reader 2	3.8±0.4	3.7±0.4	0.42	
**Image noise**				
Reader 1	3.0±0.4	3.9±0.3	<0.001	0.84
Reader 2	3.0±0.5	3.9±0.5	<0.001	
**Overall image quality**				
Reader 1	3.7±0.4	3.9±0.3	0.09	0.68
Reader 2	3.7±0.5	3.8±0.3	0.11	

* OI; original image, VI; virtual image, SDCT; standard dose CT.

### Image distinction possibility of validation set

The sensitivity and specificity for correct differentiation between SDCT in the training set and VIs were obtained by a visual assessment. Reader 1 recorded a sensitivity and specificity of 55.0% and 42.5%, respectively, and reader 2 recorded values of 67.5% and 27.5%, respectively. The overall sensitivity and specificity of the reader performance were 61.2% and 35.0%, respectively.

## Discussion

Machine learning, as a branch of artificial intelligence, has been one of the most important topics in medical imaging, and deep learning, a specific artificial neural network technique, is considered a promising type of machine learning in medical imaging [32, 33]. In this study, we adopted a deep-learning method to convert an original LDCT image into a virtual SDCT image. The results show that it is possible to train the VIGAN using an unpaired set of LDCT and SDCT images and use it for the denoising of LDCT.

In the quantitative analysis, not only the image noise of the VIs but also the CT attenuation was reduced. The CT numbers of the portal vein, liver, and paraspinal muscle were higher in the OIs than in the VIs and original SDCT. The reason for the high CT attenuation observed in the 80 kVp image can be explained as follows. The mean energy level of X-rays at 80 kVp is closer to the K-edge of iodine (33 keV) than it is at 120 kVp [34]. Hence, the mean attenuation of the vessels and organs is higher in the 80 kVp portal phase image.

However, the CNR and SNR of the portal vein and liver were higher in the VIs without a significant statistical difference. Furthermore, in the qualitative analysis, the image contrast and image noise scores of OIs were higher than those of VIs, but the overall image quality score was higher in VI. According to our quantitative and qualitative analysis, the image noise reduction is more efficient than reduced CT attenuation in VIs. Consequently, we can conclude that the proposed network is efficient in learning the differences between 80 kVp and 120 kVp images, not only for the image noise, but also for CT attenuation. In addition, the poor image distinction possibility of two readers shows that it is difficult to differentiate between SDCT and VIs only through visual assessment.

In the results of the test dataset, the mean CT numbers of VIs were lower than OIs and SAFIRE, which is similar to the results of the validation dataset. VIs of the test dataset show the lowest mean image noise and highest mean SNR among the three images (OI vs. SAFIRE vs. VI) without significant statistical difference between SAFIRE and VIs. The NPS analysis also showed that VIGAN can reduce noise while maintaining an image quality similar to that achieved with the IR method. VIGAN performs well when the test domain (80 kVp with 262 mAs) is close to the training domain (80 kVp with 100 mAs). However, further study is required to determine the robustness of the proposed deep learning based denoising method in real clinical environment. Domain adaptation techniques [35] may further improve the performance of VIGAN when the test domain is considerably different from the training domain.

Fig 4 shows that the generator loss (J(G)) of VIGAN converges (but not stably) to its local minima as the number of epochs increases. This may be related to several factors such as 1) the inherent nature of GAN (i.e., finding a solution of the min-max problem), 2) the network size and algorithms used to update the weights, and 3) the size and quality of training datasets. More rigorous analysis is needed to elucidate this phenomenon. The deep learning method was performed in a patch-by-patch manner, rather than being applied to all images. Thus, the number of training datasets was significantly increased, allowing for the efficient learning of localized noise artifacts [36]. Previously, similar approaches involving the application of GAN objective functions with additional constraints have been proposed for image-to-image translation [12, 13, 37]. The results demonstrate that the constraints used help to preserve the global structure of the input data.

The strength of our study is that we used "unpaired data sets" obtained from the CT images of real patients. Because performing a CT scan twice at the same time to prepare paired data sets is ethically unacceptable in real medical environments, it is difficult to obtain paired data sets from patients in real clinical practice. If a CT is taken to simply obtain a pair of data sets, one patient must undergo two consecutive CT scans under two protocols (80 kVp and 120 kVp). Chen et al. [8] also proposed a method for noise reduction in LDCT using a deep convolution neural network. They trained their network using the normal-dose images and the corresponding generated low-dose images. To validate the effect of the trained network, a test set was prepared by taking the chest CT of a sheep under anesthesia with two protocols: normal-dose scan (100 kVp, 150 mAs) and low-dose scan (80 kVp, 17 mAs). Suzuki et al.(9) used pairs of low-dose chest CT (0.1 mSv, 120 kVp, 4 mAs) images and corresponding high-dose CT (5.7 mSv, 120 kVp, 230 mAs) images of an anthropomorphic chest phantom reconstructed by the FBP to train their network. The trained network was applied to new LDCT (0.1 mSv) of patients from three different vendors to generate the virtual high-dose CT images. The way in which the reduction of CT radiation dose was achieved in this study was very similar to ours, except that we used unpaired data sets of real patients to train our network. We adopted a GAN framework that can be trained by using “unpaired data sets” [11–13]. There have been a few reports on the application of GANs to LDCT [14, 15]. These studies used paired data sets obtained from a phantom [14] or animal [15] to train their proposed algorithms. Yang et al. [14] compared various networks to evaluate the LDCT image denoising effect, and they proposed a network based on GAN. They used a data set authorized for “the 2016 NIH-AAPM-Mayo Clinic Low Dose CT Grand Challenge” that contained normal-dose abdominal CT images and simulated quarter-dose CT images to train and test the proposed network. Yi et al. [15] proposed a sharpness-aware GAN for LDCT denoising. They compared the GAN using two traditional image denoising methods (BM3D, K-SVD), and the images reconstructed using IR methods (ASIR and VEO) were also compared. They prepared various dose-level CT images using piglets to generate the test set. Only two patient scans without accurate dose information were used to evaluate the proposed program.

Our study has some limitations. First, the feasibility of using virtual CT images was evaluated, but the diagnostic performance was not assessed. We excluded the cases with liver abnormalities, and hence no objects were used to evaluate the diagnostic ability of the CT images. Subsequent studies on the evaluation of the diagnostic ability of virtual CT images are expected to be conducted. Second, we only compared VIGAN with SAFIRE, only one of the various types of IR software. Further comparison of different IR methods obtained from multiple centers could be required to prove the validity of VIGAN, which is a part of our future work.

In conclusion, this study shows that the deep learning method trained by unpaired datasets can improve the quality of LDCT images obtained from old CT scanner without the IR method, and can also achieve comparable image quality to the IR method. The results of our study provide a new direction for LDCT research through deep learning.

## Supporting information

S1 File Mathematical model. (DOCX)
