# Supplementary material for: Denoising of pediatric low dose abdominal CT using deep learning based algorithm
Source: PLoS One. 2022 Jan 21;17(1):e0260369. doi: 10.1371/journal.pone.0260369 (PMC8782418; doi:10.1371/journal.pone.0260369)
Supplement: S1 File — (DOCX) [file pone.0260369.s001.docx]

**Mathematical Model**

In this section, we describe a mathematical model of generating VI patches from OI patches. Let $\left\{ x_{L,i} \right\}_{i=1}^{N}$ and $\left\{ x_{H,i} \right\}_{i=1}^{M}$ be the sets of patches extracted from unpaired LDCT and SDCT images, respectively. We assume that they were drawn from an unknown LDCT patch distribution $p_{x_{L}}(x_{L}$) and a SDCT patch distribution $p_{x_{H}}(x_{H})$. Let $x_{V}$ be a SDCT-like (i.e., VI) patch corresponding to an OI patch $x_{L}$. We find the generator $G:x_{L}\to x_{V}$ satisfying the following problem:

| Eq. (1) | $G=\min_{G} {KL(p}_{G\left( x_{L} \right)},p_{x_{H}})+\lambda E_{x_{L\sim}p_{x_{L}}}[\vert\vert G\left( x_{L} \right)-x_{L}\vert\vert_{2}^{2}],$ |
| --- | --- |

where $E[x]$ denotes the expected value of $x$, $KL(p,q)$ is the Kullback-Liebler divergence between $p$ and $q$, $||\cdot||_{2}$ is the $l_{2}$ norm, and $\lambda$ is the regularization parameter. The proposed $G$ estimates the $p_{x_{H}}$ using many samples $\left\{ x_{H,i} \right\}_{i=1}^{M}$, while preserving the morphological structure of $x_{L}$ in sense of the $l_{2}$ distance.

With adversarial loss, Eq. (1) can be expressed as a GAN framework:

| Eq. (2) | $G=\min_{G} J\left( G \right):=\min_{G} E_{x_{L\sim}p_{x_{L}}}[log(1-D\left( G\left( x_{L} \right) \right)+\lambda\vert\vert G\left( x_{L} \right)-x_{L}\vert\vert_{2}^{2}],$ |
| --- | --- |

where the optimal discriminator $D$ is given by

| Eq. (3) | $D=\min_{D} E_{x_{H\sim}p_{x_{H}}}[D\left( x_{H} \right)]+E_{x_{L\sim}p_{x_{L}}}[log(1-D\left( G\left( x_{L} \right) \right)]$ |
| --- | --- |

The derivation of Eq. (2) is described in a previous paper [18]. In the proposed model, no loss function between the LDCT images and the corresponding SDCT images is required. Hence, it is possible to perform training with unpaired LDCT and SDCT datasets. The problem of Eqs. (2) and (3) can be solved by using the standard backpropagation method provided in TensorFlow.
